# Efficacy and influencing factors of rituximab in idiopathic membranous nephropathy: A real-world retrospective study

**DOI:** 10.1097/MD.0000000000051442

**Published:** 2026-10-30

**Authors:** Xiaofei Xu, Qiwei Zhou, Subo Li, Xiaoru Zhang, Xiaoyun Zhang

**Affiliations:** a Department of Nephrology, The First People’s Hospital of Xiaoshan District, Hangzhou, Zhejiang Province, China; b Medical Device Inspection Section, Zhejiang Center for Drug Inspection, Hangzhou, Zhejiang Province, China.

**Keywords:** efficacy, idiopathic membranous nephropathy, rituximab

## Abstract

This study aims to investigate the efficacy and influencing factors of rituximab (RTX) in the treatment of idiopathic membranous nephropathy (IMN). A total of 46 patients with IMN visited and followed up regularly in our hospital were included in this study. The patients were divided into 2 groups, with 23 patients receiving RTX treatment (RTX group) and 23 patients receiving treatment with cyclophosphamide (CTX group). The clinical data of the patients, including urine examination, blood test, efficacy evaluation results, and adverse events (AEs) were analyzed retrospectively. The changes in clinical and biochemical indicators were compared in RTX groups before and after treatment, and the clinical efficacy and influencing factors of RTX treatment for IMN were evaluated. After 6 months of treatment, in the RTX group, compared to baseline, there was a significant decrease in 24-hour urine protein, total cholesterol, and B lymphocyte count (*P* < .001, *P* = .010, *P* < .001), while albumin (ALB), globulin, and natural killer cells showed a significant increase compared to baseline (*P* < .001, *P* < .001, *P* < .001). After 6 months of treatment, the RTX group showed significantly higher glomerular filtration rate, higher levels of ALB, and higher levels of globulin compared to the CTX group, with statistical significance (*P* = .042, *P* = .003, *P* < .001). Among patients with positive anti-PLA2R antibodies at baseline, the antibody clearance rate at 6 months was significantly higher in the RTX group than in the CTX group (*P* = .035). By the end of follow-up, the overall remission rates were 91.3% for the RTX group and 87.0% for the CTX group, with no statistically significant difference between the 2 groups (*P* > .99). A univariate analysis of the correlation between clinical characteristics, laboratory tests, and the efficacy of RTX treatment found that lower baseline 24-hour urine protein levels appeared to be associated with a higher likelihood of achieving remission (*P* = .035). AEs were observed in 17.4% of RTX-treated patients versus 52.2% of CTX-treated patients (*P* = .030). RTX appeared to be effective in patients with IMN and was associated with a more favorable safety profile compared with CTX.

## 1. Introduction

Idiopathic membranous nephropathy (IMN) is a leading cause of end-stage kidney disease, characterized by a complex and diverse clinical presentation, leading to variable prognostic outcomes. Around 20 to 25% of patients with IMN can spontaneously enter a remission phase, while about 40% of patients progress to end-stage kidney disease within 10 years. Glucocorticoids and cyclophosphamide (CTX) therapy is the first-line treatment regimen for membranous nephropathy. Research has indicated that this combined treatment is effective in 60 to 70% of patients.

However, CTX is associated with risks such as bone marrow suppression, gonadal toxicity, and potential carcinogenic effects.^[1]^ Though calcineurin inhibitors, including cyclosporine and tacrolimus, induce remission in approximately 70% of patients with nephrotic syndrome, their widespread use is limited by a high relapse rate following treatment discontinuation.^[2]^

Rituximab (RTX), a monoclonal antibody targeting the B-cell surface marker CD20, effectively depletes B cells, disrupts antibody production, and interferes with the pathogenesis of IMN.^[3]^ RTX has been shown to significantly reduce B cell counts and levels of anti-PLA2R antibodies in patients with membranous nephropathy, providing a strong theoretical foundation for its application in treating IMN. RTX demonstrates a greater efficacy than conservative treatments in achieving remission and has shown non-inferior outcomes compared to cyclosporine. Compared to CTX therapy, RTX may also have a more favorable safety profile, but there is a lack of comparison between RTX and CTX therapy. Given the substantial toxicity burden associated with long-term CTX use – including bone marrow suppression, gonadal dysfunction, and secondary malignancy – there is a pressing clinical need to determine whether RTX can serve as a safer yet equally efficacious alternative.^[4,5]^ Preliminary data from the RI-CYCLO randomized trial suggest comparable efficacy between RTX and CTX; however, further evidence derived from real-world clinical practice remains warranted.^[6]^ In China, CTX continues to be widely employed as a first-line immunosuppressive agent, yet comprehensive comparative studies evaluating RTX against CTX monotherapy remain scarce. Differences in patient characteristics, anti-PLA2R antibody positivity status, and proteinuria severity may all exert a substantial influence on treatment outcomes in real-world clinical settings. Furthermore, the identification of reliable predictors of RTX response remains an unresolved issue of considerable clinical importance, with direct implications for patient selection and the optimization of individualized therapeutic strategies. This study aims to evaluate the clinical efficacy of RTX compared to CTX in IMN patients, to analyze the therapeutic effects of RTX in clinical practice, and identify factors that may influence treatment outcomes.

## 2. Materials and methods

### 2.1. Research subjects

For this study, we included IMN patients who received treatment with RTX or the combination of glucocorticoids and CTX at the First People’s Hospital of Xiaoshan District from January 2022 to December 2023.

Inclusion criteria were as follows: Age 18 years or older; Diagnosis of IMN confirmed via renal biopsy; and Regular follow-up and availability of complete medical records.

Exclusion criteria included: Secondary membranous nephropathy; Patients with severe infections or severe mental illnesses; Patients with concomitant tumors; Prior use of RTX, CTX, or other immunosuppressants; and Pregnant women.

All treatment decisions were made by the attending nephrologists in accordance with current clinical guidelines and patient preference. Patients were followed up from the commencement of medication, with follow-up carried out through review of medical records and telephone interviews. The follow-up period for all patients concluded on June 20, 2024, with a minimum follow-up duration of 6 months. This research project was designed in accordance with the Helsinki Declaration and received approval from the Ethics Committee of the First People’s Hospital of Xiaoshan District. Participants provided informed consent for publication of study findings.

### 2.2. Treatment regimen

During the course of treatment, patients were advised to follow a diet low in salt and fat while rich in high-quality protein, with a daily sodium intake restricted to <4 grams. Concurrently, patients received antihypertensive therapy, including renin-angiotensin system inhibitors in combination with calcium channel blockers and other medications, to control blood pressure, reduce cholesterol levels, provide anticoagulation, and manage related symptoms appropriately.

#### 2.2.1. RTX group

Patients received RTX as treatment, with an initial intravenous infusion of 1 gram of RTX, followed by another 1 gram infusion 2 weeks later; or intravenous infusions of RTX at a dosage of 375 mg/m^2^, administered 4 times with a one-week interval between each dose. The choice of regimen was determined by the treating nephrologist based on individual patient characteristics, body surface area, and clinical judgment, consistent with both regimens being recommended by current KDIGO 2021 guidelines.

#### 2.2.2. CTX group

Patients underwent treatment with CTX in an alternating six-month cycle, with CTX administered every month. Simultaneously, oral prednisone tablets (produced by Zhejiang Xianju Pharmaceutical Co., Ltd., Taizhou, Zhejiang Province, China) were taken at a dosage of 1.0 mg/(kg·d), gradually tapered off over 8 to 12 weeks after initiation.

### 2.3. Observation and follow-up indicators

General information: gender, age. Laboratory tests: serum creatinine, blood urea nitrogen, estimated glomerular filtration rate (eGFR), 24-hour urine protein quantification, blood uric acid, plasma albumin (ALB), globulin, cholesterol, triglycerides, anti-PLA2R antibody, total T lymphocyte count, CD4^+^ lymphocytes, CD8^+^ lymphocytes, CD4^+^CD8^+^ double positive cells, natural killer cell count, B lymphocyte count. eGFR was calculated using the CKD-EPI equation to assess renal function.^[7]^ Follow-up endpoints include mortality, maintenance hemodialysis, and progression to end-stage renal disease (defined as eGFR < 15 mL/min sustained for more than 3 months). Efficacy assessment: Complete remission (CR): urine protein (Upro) < 0.3 grams/day, ALB > 30 grams/L, normal renal function; PR: Upro = 0.3–3.5 grams/day, decreased by more than 50% compared to before treatment, ALB ≥ 30 grams/L, stable renal function; Ineffective: Upro decreased by <50% compared to before treatment, ALB < 30 grams/L, or worsening renal function; Relapse: Patients who achieved CR or PR experience a reappearance of 24-hour urine protein quantification exceeding 50% of pretreatment levels or exceeding 3.5 grams; and Progression: Serum creatinine increases by more than 30% above baseline, or progresses to end-stage chronic kidney disease with eGFR ≤ 15 mL/min. Remission rate = (number of complete remissions + number of partial remissions)/total number of cases × 100%.

### 2.4. Safety assessment

All treatment-related adverse events (AEs) were systematically recorded for all patients in both groups, including infusion-related reactions, infections (upper respiratory tract infection, pulmonary infection, and urinary tract infection), hepatic dysfunction, hyperglycemia, and hypertension.

### 2.5. Statistical analysis

Statistical analysis was conducted using SPSS version 26.0 and GraphPad Prism 8 for data processing. Categorical covariates were reported as percentages and counts. Continuous variables were reported as medians and ranges. Continuous data following a normal distribution were analyzed using *t*-tests or one-way analysis of variance (ANOVA). For non-normally distributed data, values were analyzed using the Mann–Whitney *U* test. Differences in categorical data were evaluated with the chi-square test. A *P*-value of <.05 was considered statistically significant.

## 3. Results

### 3.1. Comparison of baseline data between the 2 groups of patients with IMN

A total of 46 patients were included in this study, with 23 patients receiving treatment with RTX categorized as the RTX group, and 23 patients receiving treatment with CTX categorized as the CTX group. The median age at presentation was 54.5 years (range, 32–85 years). In the RTX group, there were 11 males and 12 females. In the CTX group, there were 13 males and 10 females. The median follow-up time for the patients was 13 months (interquartile range, 6–30 months). There were no statistically significant differences in age, gender, pretreatment serum creatinine, blood urea nitrogen, eGFR, 24-hour urine protein quantification, blood uric acid, plasma ALB, globulin, cholesterol, triglycerides and anti-PLA2R antibody positivity between the 2 groups (*P* > .05) (Table 1).

**Table 1:** Comparison of baseline data between the 2 groups of patients with idiopathic membranous nephropathy.

Parameters	RTX group (n = 23)	CTX group (n = 23)	*P*-value
Gender (male/female)	11/12	13/10	.768
Age (yr)	52 (32–83)	59 (35–85)	.117
Creatinine (μmol/L)	75.2 (45.1–165.1)	77.6 (44.2–122.3)	.939
Urea nitrogen (mmol/L)	5.56 (3.00–55.00)	6.21 (3.52–22.25)	.660
eGFR (mL/min)	91 (41–134)	80 (47–128)	.721
24 h urine protein quantification (g/d)	5.82 (1.93–18.98)	4.34 (0.48–16.50)	.396
Uric acid (μmol/L)	364 (184–538)	409 (222–644)	.106
Albumin (g/L)	24.1 (11.7–35.9)	23.2 (15.3–36.4)	.720
Globulin (g/L)	20.6 (14.7–30.2)	21.9 (17.5–36.5)	.260
Cholesterol (mmol/L)	6.57 (4.54–13.38)	7.14 (2.30–14.40)	.816
Triglycerides (mmol/L)	2.37 (0.82–6.65)	1.59 (0.68–8.85)	.164
Anti-PLA2R antibody positivity, n (%)	18 (78.26%)	18 (78.26%)	>.99

CTX = cyclophosphamide, eGFR = estimated glomerular filtration rate, RTX = rituximab.

### 3.2. Comparison of RTX group between 6 months post-treatment and baseline

At 6 months post-treatment, the RTX group demonstrated significant reductions in 24-hour urine protein, total cholesterol, and B lymphocyte count compared to baseline (*P* < .001, *P* = .010, *P* < .001). Conversely, levels of ALB, globulin, and natural killer cells showed significant increases from baseline (*P* < .001 for all) (Table 2).

**Table 2:** Comparison of rituximab group between 6 mo post-treatment and baseline.

Parameters	Before therapy (n = 23)	After therapy (n = 23)	*P*-value
Creatinine (μmol/L)	75.2 (45.1–165.1)	74.60 (41.60–121.70)	.318
Urea nitrogen (mmol/L)	5.56 (3.00–55.00)	5.89 (3.94–10.37)	.318
eGFR (mL/min)	91 (41–134)	95 (45–129)	.509
24 h urine protein quantification (g/d)	5.82 (1.93–18.98)	0.65 (0.03–8.44)	<.001
Uric acid (μmol/L)	364 (184–538)	342 (186–689)	.936
Albumin (g/L)	24.1 (11.7–35.9)	42.5 (30.0–45.7)	<.001
Globulin (g/L)	20.6 (14.7–30.2)	25.7 (21.2–31.7)	<.001
Cholesterol (mmol/L)	6.57 (4.54–13.38)	5.69 (3.90–9.74)	.010
Triglycerides (mmol/L)	2.37 (0.82–6.65)	1.93 (0.79–21.00)	.061
Total T lymphocytes (M/L)	1180 (542–2428)	1266 (477–3037)	.821
CD4^+^ T lymphocytes (M/L)	714 (267–1845)	798 (272–2134)	.402
CD8^+^ T lymphocytes (M/L)	418 (182–1222)	402 (173–805)	.956
CD4^+^ CD8^+^ T lymphocytes (M/L)	6 (1–22)	7 (1–22)	.842
Natural killer cell (M/L)	149 (42–599)	460 (81–983)	<.001
B lymphocytes (M/L)	229 (12–853)	0 (0–72)	<.001

eGFR = estimated glomerular filtration rate.

### 3.3. Comparison of efficacy between the 2 groups at 6 months

After 6 months of treatment the RTX group exhibited significantly higher eGFR [95 (45–129) vs 77 (41–122) mL/min, *P* = .042], ALB levels [42.5 (30.0–45.7) vs 36.7 (25.6–44.5) grams/L, *P* = .003], and globulin levels [25.7 (21.2–31.7) vs 21.4 (17.1–26.6) grams/L, *P* < .001] compared to the CTX group, with statistical significance noted (Table 3, Fig. 1C, F, and G). No statistically significant differences were found in serum creatinine, blood urea nitrogen, 24-hour urine protein quantification, blood uric acid, cholesterol, and triglycerides between the 2 groups (Table 3, Fig. 1A, B, D, E, H, and I).

**Table 3:** Comparison of efficacy between the 2 groups at 6 mo.

Parameters	RTX group (n = 23)	CTX group (n = 23)	*P*-value
Creatinine (μmol/L)	74.60 (41.60–121.70)	89.00 (63.60–149.50)	.063
Urea nitrogen (mmol/L)	5.89 (3.94–10.37)	6.54 (3.49–11.26)	.069
eGFR (mL/min)	95 (45–129)	77 (41–122)	.042
24 h urine protein quantification (g/d)	0.65 (0.03–8.44)	0.54 (0.10–4.67)	.784
Uric acid (μmol/L)	342 (186–689)	374 (215–502)	.545
Albumin (g/L)	42.5 (30.0–45.7)	36.7 (25.6–44.5)	.003
Globulin (g/L)	25.7 (21.2–31.7)	21.4 (17.1–26.6)	<.001
Cholesterol (mmol/L)	5.69 (3.90–9.74)	5.63 (4.12–8.73)	.757
Triglycerides (mmol/L)	1.93 (0.79–21.00)	1.61 (0.72–3.29)	.064

CTX = cyclophosphamide, eGFR = estimated glomerular filtration rate, RTX = rituximab.

**Figure 1.**
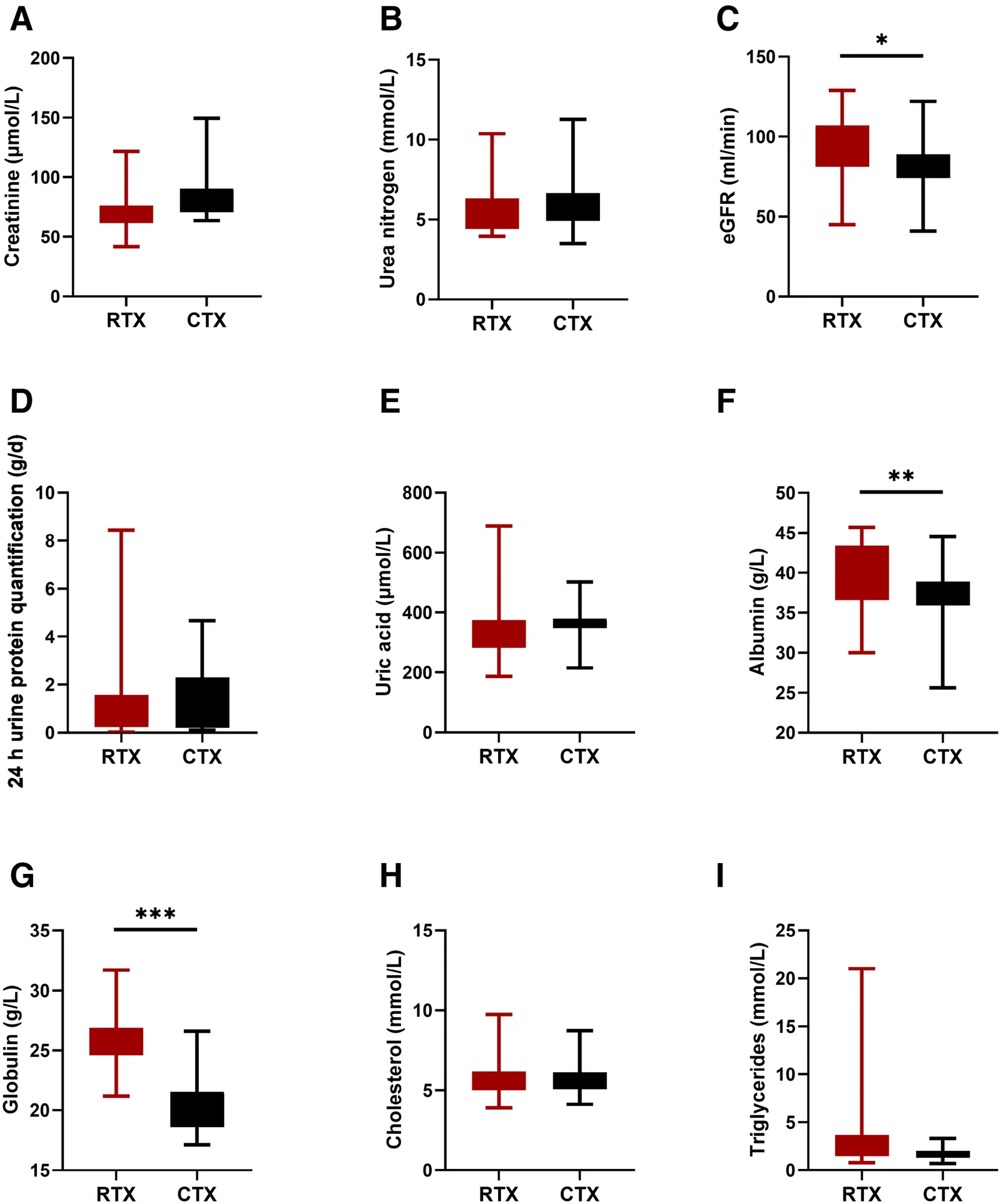
Comparison of efficacy between the 2 groups at 6 mo. (A) Creatinine. (B) Urea nitrogen. (C) eGFR. (D) 24 h urine protein quantification. (E) Uric acid. (F) Albumin. (G) Globulin. (H) Cholesterol. (I) Triglycerides. CTX = cyclophosphamide, eGFR = estimated glomerular filtration rate, RTX = rituximab. *P < .05; **P < .01; ***P < .001.

### 3.4. Comparison of anti-PLA2R antibody clearance rate between the 2 groups at 6 months

Among patients with positive anti-PLA2R antibodies at baseline, the antibody clearance rate after 6 months of treatment was significantly higher in the RTX group than in the CTX group [55.56% (10/18) vs 16.67% (3/18), *P* = .035], indicating that RTX more effectively promoted anti-PLA2R antibody clearance compared to CTX.

### 3.5. Comparison of the overall remission rates between the 2 groups

After 6 months of treatment, in the RTX group, 9 cases achieved CR and 11 cases achieved PR, with a total remission rate of 87.0%. In the CTX group, 8 cases achieved CR, 11 cases achieved PR, and the total remission rate was 82.6%. There was no statistically significant difference in the total remission rate between the 2 groups (*P* > .99) (Fig. 2A). During the follow-up period, the RTX group achieved 11 cases of CR and 10 cases of PR, resulting in an overall remission rate of 91.3%. In the CTX group, there were 13 cases of CR and 7 cases of PR, resulting in an overall remission rate of 87.0%. The difference in overall remission rates between the 2 groups was not statistically significant (*P* > .99) (Fig. 2B). Although no statistically significant difference in remission rates was detected between the 2 groups, both regimens demonstrated clinically meaningful remission rates exceeding 80%, suggesting comparable therapeutic efficacy in inducing remission in patients with membranous nephropathy.

**Figure 2.**
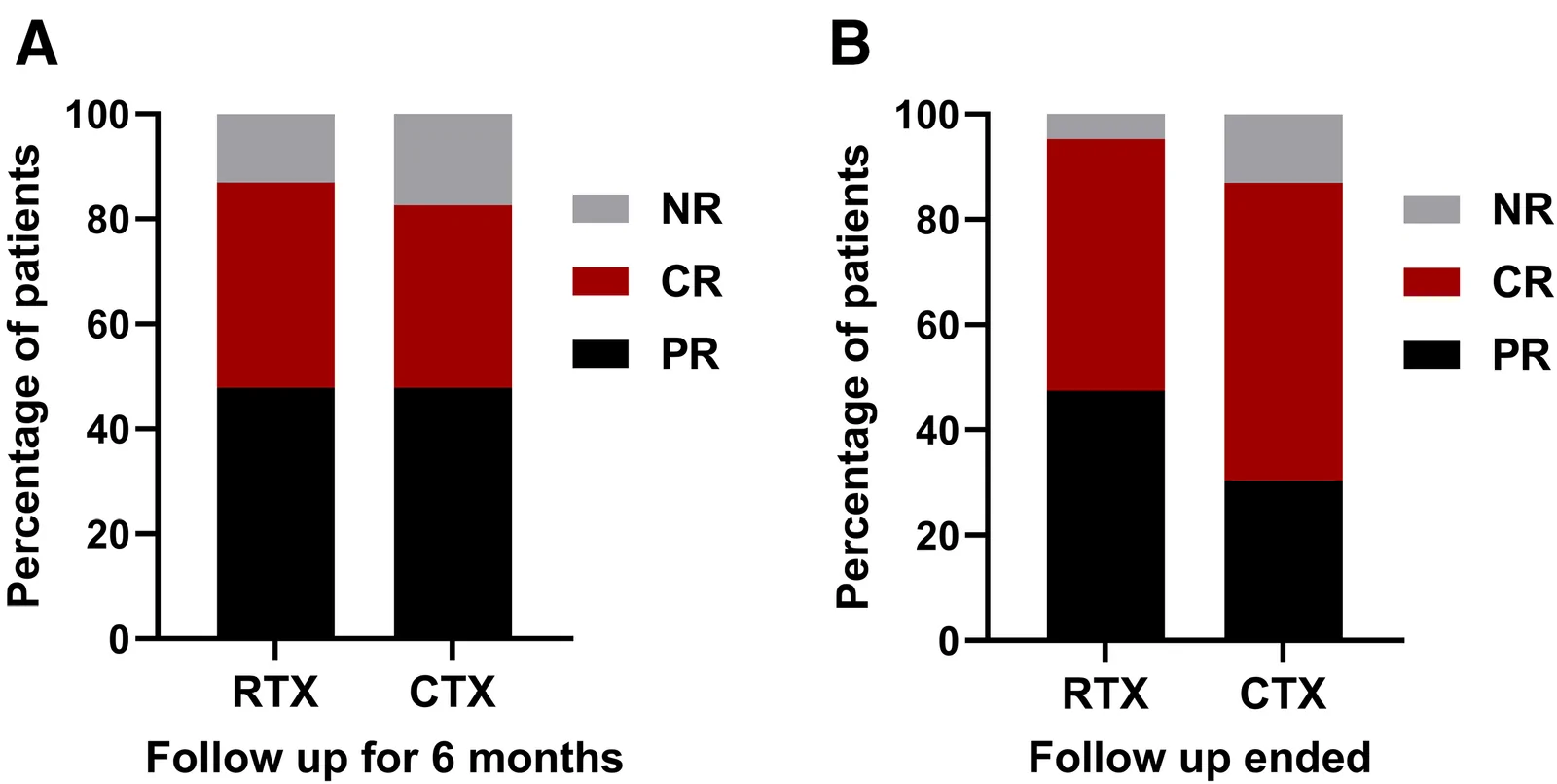
The efficacy outcome between the 2 groups. (A) In the 6-mo follow-up. (B) The end of follow-up. CR = complete remission, CTX = cyclophosphamide, NR = no response, PR = partial remission, RTX = rituximab.

### 3.6. Univariate analysis of factors associated with RTX treatment

By the end of the follow-up, 21 patients in the RTX group achieved remission, while 2 patients did not. In this exploratory univariate analysis, the achievement of remission appeared to be associated with baseline 24-hour urine protein levels (*P* = .035), with patients achieving remission demonstrating notably lower baseline proteinuria than those who did not (5.79 vs 13.25 grams/day) (Table 4). Given that only 2 patients were in the no-remission group, this finding should be regarded as a preliminary signal rather than an established basis for patient selection, and it warrants further validation in larger, prospective studies.

**Table 4:** Univariate analysis of the correlation of rituximab efficacy.

Parameters	Remission group (n = 21)	No-remission group (n = 2)	*P*-value
Gender (male/female)	10/11	1/1	>.99
Age (yr)	52 (32–83)	61.50 (52–71)	.442
Creatinine (μmol/L)	74.7 (45.1–120.4)	120.5 (75.9–165.1)	.518
Urea nitrogen (mmol/L)	5.56 (3.00–55.00)	7.68 (4.33–11.03)	.957
eGFR (mL/min)	96 (44–134)	54.5 (41–68)	.081
24 h urine protein quantification (g/d)	5.79 (1.93–16.32)	13.25 (7.51–18.98)	.035
Uric acid (μmol/L)	364 (184–538)	346.50 (265–428)	.998
Albumin (g/L)	24.1 (11.7–35.9)	19.8 (14.0–25.6)	.410
Globulin (g/L)	20.6 (14.7–30.2)	23.45 (20.5–26.4)	.460
Cholesterol (mmol/L)	6.57 (4.54–13.38)	6.84 (5.43–8.24)	.822
Triglycerides (mmol/L)	2.17 (0.82–6.65)	3.73 (3.57–3.88)	.301
Anti-PLA2R antibody positivity, n (%)	16 (76.19%)	2 (100%)	>.99
Total T lymphocytes (M/L)	1180 (542–2428)	1347 (982–1712)	.931
CD4^+^ T lymphocytes (M/L)	714 (267–1845)	1007 (698–1316)	.663
CD8^+^ T lymphocytes (M/L)	424 (182–1222)	372.5 (362–383)	.659
CD4^+^ CD8^+^ T lymphocytes (M/L)	6 (1–22)	10 (5–15)	.521
Natural killer cell (M/L)	149 (42–599)	358.5 (132–585)	.556
B lymphocytes (M/L)	229 (12–853)	306 (220–392)	.859

eGFR = estimated glomerular filtration rate.

### 3.7. Safety analysis: comparison of adverse events

In the RTX group, AEs were observed in 4 of 23 patients (17.4%). The predominant adverse event was infusion-related reactions, occurring in 3 patients (13.0%), manifesting as throat discomfort, chills, fever, and skin rash following the first infusion; all symptoms resolved promptly after administration of corticosteroids or antihistamines. One patient (4.3%) developed an upper respiratory infection, which resolved following standard anti-infective therapy.

In the CTX group, AEs were recorded in 12 of 23 patients (52.2%), comprising infections in 4 patients (17.4%; including 1 case of upper respiratory infection, 2 cases of pulmonary infection, and 1 case of urinary tract infection), liver dysfunction in 4 patients (17.4%), hyperglycemia in 3 patients (13.0%), and hypertension in 1 patient (4.3%).

The overall adverse event incidence rate was significantly lower in the RTX group compared to the CTX group (17.4% vs 52.2%, *P* = .030) (Table 5), indicating that RTX was associated with a more favorable safety profile than CTX in the treatment of IMN.

**Table 5:** Comparison of adverse events between the 2 groups.

Type of adverse event	RTX group (n = 23)	CTX group (n = 23)	*P*-value
Infusion-related reaction	3 (13.0%)	0 (0%)	
Infection	1 (4.3%)	4 (17.4%)	
Upper respiratory infection	1 (4.3%)	1 (4.3%)	
Pulmonary infection	0 (0%)	2 (8.7%)	
Urinary tract infection	0 (0%)	1 (4.3%)	
Liver dysfunction	0 (0%)	4 (17.4%)	
Hyperglycemia	0 (0%)	3 (13.0%)	
Hypertension	0 (0%)	1 (4.3%)	
Total adverse events	4 (17.4%)	12 (52.2%)	.030

CTX = cyclophosphamide, RTX = rituximab.

## 4. Discussion

According to the KDIGO guidelines, initial treatment for patients with intermediate to high-risk IMN may involve the use of RTX as a standalone therapy. Additionally, for patients who have previously been treated with other immunosuppressive agents and have experienced relapse or refractory disease, RTX therapy is also recommended. This suggests that RTX may have a beneficial therapeutic effect in these specific patient populations, serving as a valuable treatment option for improving clinical outcomes and controlling disease activity.

The results of this study demonstrate that patients who received rituximab treatment experienced significant reductions in 24-hour urine protein levels, total cholesterol, and B lymphocyte counts, while showing significant increases in ALB, globulin, and natural killer cell levels compared to pretreatment values. Consistent with findings from other studies, this research indicated that after RTX treatment, there were persistent changes in B cell subpopulations and a significant increase in NK cells.^[8]^ Several observational studies have reported on the safety and efficacy of RTX in patients with IMN. These studies collected data on 24-hour urine protein and ALB levels before and 6 months after RTX treatment, demonstrating that RTX effectively reduced proteinuria and increased serum ALB levels.^[9]^ Furthermore, another study found that RTX can also reduce cholesterol levels in patients.^[10]^

In this study, we compared the efficacy of RTX with the CTX regimen in patients with IMN. The results indicate that, compared to the CTX group, the RTX group had higher levels of ALB, globulin, and glomerular filtration rate 6 months after treatment. However, there was no statistically significant difference in the overall remission rate between the 2 groups, which aligns with findings from another retrospective study that compared the efficacy and safety of RTX with the CTX regimen, showing similar remission rates and significantly fewer AEs in the RTX group.^[11]^ The study reported that RTX demonstrated efficacy comparable to the CTX regimen in the treatment of membranous nephropathy.^[6]^

Beyond the differences in clinical parameters, the present study demonstrated that RTX more effectively promoted anti-PLA2R antibody clearance compared to CTX, with a significantly higher clearance rate in the RTX group. This finding is mechanistically consistent with RTX’s selective depletion of CD20-positive B lymphocytes, which are the primary source of anti-PLA2R antibody production, thereby more directly targeting the autoimmune pathogenesis of IMN.^[5]^ In contrast, CTX acts through a broader cytotoxic mechanism that may be less efficient at suppressing disease-specific autoantibody production. Notably, despite the superior immunological response in the RTX group, overall clinical remission rates between the 2 groups were comparable, suggesting that antibody clearance may serve as an earlier surrogate marker of treatment response, preceding clinical remission.

Although long-term evidence regarding reduction in kidney failure and mortality risk is more established for CTX-based regimens, the cumulative toxicity of CTX remains a major concern.^[1]^ Due to the absence of an established safe threshold for cumulative CTX doses linked to carcinogenic and gonadotoxic effects, its use is recommended in patients at higher risk of renal function decline. Considering the comparable efficacy of both treatment options, the more targeted immunological response evidenced by superior anti-PLA2R antibody clearance observed in the RTX group, and the tendency for IMN to relapse, RTX may be considered as a first-line therapy for recurrent treatment of IMN.

However, RTX may not be the optimal choice for all patients. Another study indicates that 20 to 40% of patients do not respond to RTX.^[1]^ There are also varying proportions of patients who are intolerant to RTX yet respond favorably to CTX regimens. Identifying patients who may be resistant to RTX or have a poorer prognosis remains challenging. Our findings suggest that patients with lower baseline 24-hour urine protein levels were more likely to achieve remission post-RTX treatment. This preliminary observation is directionally consistent with a prior study, which also reported a correlation between baseline urine protein levels and RTX response in IMN patients,^[12]^ lending contextual support to this exploratory finding. Patients with higher baseline proteinuria may warrant closer clinical monitoring. By utilizing clinical and serological markers – including proteinuria, renal function, ALB levels, and potentially future genetic markers – we may enhance our ability to stratify patients with IMN for risk assessment and inform treatment decisions.^[6]^ Additionally, methods based on big data and machine learning may provide new insights for identifying patients who respond poorly to RTX. These research directions imply that in personalized medicine, future strategies can be developed more precisely to improve patient outcomes.

Regarding safety, the RTX-based regimen demonstrated a more favorable safety profile than CTX. Events in the RTX group were predominantly mild infusion-related reactions that resolved, whereas the CTX group experienced a broader spectrum of complications, including infections, liver dysfunction, hyperglycemia, and hypertension. These findings suggest that RTX may offer a safer therapeutic option for the long-term management of IMN.

Several limitations of this study should be acknowledged. First, the retrospective and non-randomized design introduces potential selection bias, which may confound comparisons between groups. Furthermore, the use of 2 RTX dosing regimens, both recommended by KDIGO 2021 guidelines, nonetheless introduces pharmacological heterogeneity that may have influenced treatment outcomes; a formal subgroup comparison between the 2 regimens was not feasible given the limited sample size. Second, the relatively small sample size may have limited statistical power to detect differences between treatment groups, particularly for secondary outcomes. Third, the analysis of predictors of RTX response was based on a no-remission group of only 2 patients, which severely constrains the reliability and generalizability of these findings, and no multivariate adjustment was performed due to the insufficient sample size. Well-designed prospective, randomized controlled trials are needed to confirm and build upon the associations observed in the present study.

In conclusion, RTX has demonstrated efficacy in the treatment of patients with IMN. In this exploratory analysis, patients exhibiting lower levels of 24-hour urine protein prior to treatment appeared more likely to achieve remission with RTX therapy. Our findings of RTX’s efficacy and its influencing factors provide a preliminary real-world evidence base for clinical practice and may contribute to improving treatment outcomes and long-term prognosis for patients with IMN.

## Author contributions

**Conceptualization:** Xiaofei Xu.

**Data curation:** Xiaofei Xu.

**Formal analysis:** Qiwei Zhou.

**Funding acquisition:** Xiaoru Zhang.

**Investigation:** Subo Li.

**Methodology:** Subo Li.

**Supervision:** Qiwei Zhou.

**Validation:** Xiaoyun Zhang.

**Visualization:** Xiaoyun Zhang.

**Writing – original draft:** Xiaofei Xu.

**Writing – review & editing:** Xiaofei Xu.

## References

[1] Fervenza FC Appel GB Barbour SJ; MENTOR Investigators. Rituximab or cyclosporine in the treatment of membranous nephropathy. N Engl J Med. 2019;381:36–46. 10.1056/NEJMoa1814427 31269364

[2] Fernández-Juárez G Rojas-Rivera J Logt AV; STARMEN Investigators. The STARMEN trial indicates that alternating treatment with corticosteroids and cyclophosphamide is superior to sequential treatment with tacrolimus and rituximab in primary membranous nephropathy. Kidney Int. 2021;99:986–98. 10.1016/j.kint.2020.10.014 33166580

[3] Tesar V Hruskova Z. Autoantibodies in the diagnosis, monitoring, and treatment of membranous nephropathy. Front Immunol. 2021;12:593288. 10.3389/fimmu.2021.593288 PMC8019786 33828546

[4] Kidney Disease: Improving Global Outcomes (KDIGO) Glomerular Diseases Work Group. KDIGO 2021 clinical practice guideline for the management of glomerular diseases. Kidney Int. 2021;100:S1–S276. 10.1016/j.kint.2021.05.021 34556256

[5] Huang J Yang Y Wang Y. Effectiveness and safety of rituximab monotherapy versus conventional regimens for adult idiopathic membranous nephropathy: real-world retrospective study. Front Immunol. 2025;16:1671251. 10.3389/fimmu.2025.1671251 PMC12643969 41306981

[6] Scolari F Delbarba E Santoro D; RI-CYCLO Investigators. Rituximab or cyclophosphamide in the treatment of membranous nephropathy: the RI-CYCLO randomized trial. J Am Soc Nephrol. 2021;32:972–82. 10.1681/ASN.2020071091 PMC8017548 33649098

[7] Levey AS Stevens LA Schmid CH; CKD-EPI (Chronic Kidney Disease Epidemiology Collaboration). A new equation to estimate glomerular filtration rate. Ann Intern Med. 2009;150:604–12. 10.7326/0003-4819-150-9-200905050-00006 PMC2763564 19414839

[8] Rosenzwajg M Languille E Debiec H. B- and T-cell subpopulations in patients with severe idiopathic membranous nephropathy may predict an early response to rituximab. Kidney Int. 2017;92:227–37. 10.1016/j.kint.2017.01.012 28318628

[9] Angioi A Lepori N López AC Sethi S Fervenza FC Pani A. Treatment of primary membranous nephropathy: where are we now? J Nephrol. 2018;31:489–502. 10.1007/s40620-017-0427-5 28875476

[10] Chen P Mao M Wang C. Preliminary study on the efficacy of rituximab in the treatment of idiopathic membranous nephropathy: a single-centre experience. Front Endocrinol (Lausanne). 2023;14:1044782. 10.3389/fendo.2023.1044782 PMC9974647 36875477

[11] van den Brand JAJG Ruggenenti P Chianca A. Safety of rituximab compared with steroids and cyclophosphamide for idiopathic membranous nephropathy. J Am Soc Nephrol. 2017;28:2729–37. 10.1681/ASN.2016091022 PMC5576929 28487395

[12] Irazabal MV Eirin A Lieske J. Low- and high-molecular-weight urinary proteins as predictors of response to rituximab in patients with membranous nephropathy: a prospective study. Nephrol Dial Transplant. 2013;28:137–46. 10.1093/ndt/gfs379 PMC3616763 22987142

